# Giant True Brachial Artery Aneurysm: A Case Report and Comprehensive Literature Review

**DOI:** 10.7759/cureus.94393

**Published:** 2025-10-12

**Authors:** Dimitrios A Chatzelas, Vasiliki-Elisavet P Stratinaki, Georgios V Tsamourlidis, Theodosia N Zampaka, Ioanna I Kiose, Georgios A Pitoulias, Apostolos G Pitoulias

**Affiliations:** 1 2nd Department of Surgery, Division of Vascular Surgery, “G. Gennimatas” General Hospital, Faculty of Medicine, Aristotle University of Thessaloniki, Thessaloniki, GRC; 2 Department of Vascular and Endovascular Surgery, St. Bernward Hospital, Hildesheim, DEU

**Keywords:** brachial artery aneurysm, case report, open surgery, peripheral artery aneurysm, upper extremity aneurysm

## Abstract

True brachial artery aneurysms (BAAs) constitute an extremely rare vascular entity. We present the case of a 68-year-old man with a giant, secondary, symptomatic, saccular, true BAA of the right, dominant arm, measuring 58×55mm. Open surgery was performed under general anesthesia, with aneurysm sac resection and arterial reconstruction with interposition of a reversed great saphenous vein graft. There were no postoperative complications. The patient remains up to date under regular follow-up. The histological analysis was consistent with a true degenerative aneurysm. A comprehensive review of the literature was conducted, highlighting current knowledge on true BAA pathogenesis, clinical features, differential diagnosis, imaging modalities, and treatment strategies.

## Introduction

Brachial artery aneurysms (BAAs) have been defined as isolated, focal dilatation of the brachial artery of at least 50% increase in its diameter, compared to its average diameter, adjusted for age, gender, and race [1]. True BAA pathology involves all three layers of the vessel wall, as opposed to false or pseudoaneurysms, which comprise the vast majority of BAA [2]. True BAAs are extremely rare, accounting in the literature for only 0.17% of all peripheral artery aneurysms [3]. Their natural course is unknown, and data regarding demographics, risk factors, diagnosis, and treatment strategies are scarce, due to the rarity of this vascular pathology [3].

We report a case of a 68-year-old man with a giant, true BAA of the dominant arm, which was treated by open surgery in our department. Moreover, we performed a comprehensive literature review in order to sum up the contemporary knowledge on this extremely rare pathology. The study protocol received approval from the institutional review board (approval no. 45/2023) and adhered to the principles outlined in the Helsinki Declaration (2013 amendment). The patient provided written informed consent for the operation and the publication of his medical information and images.

## Case presentation

**Figure 1:**
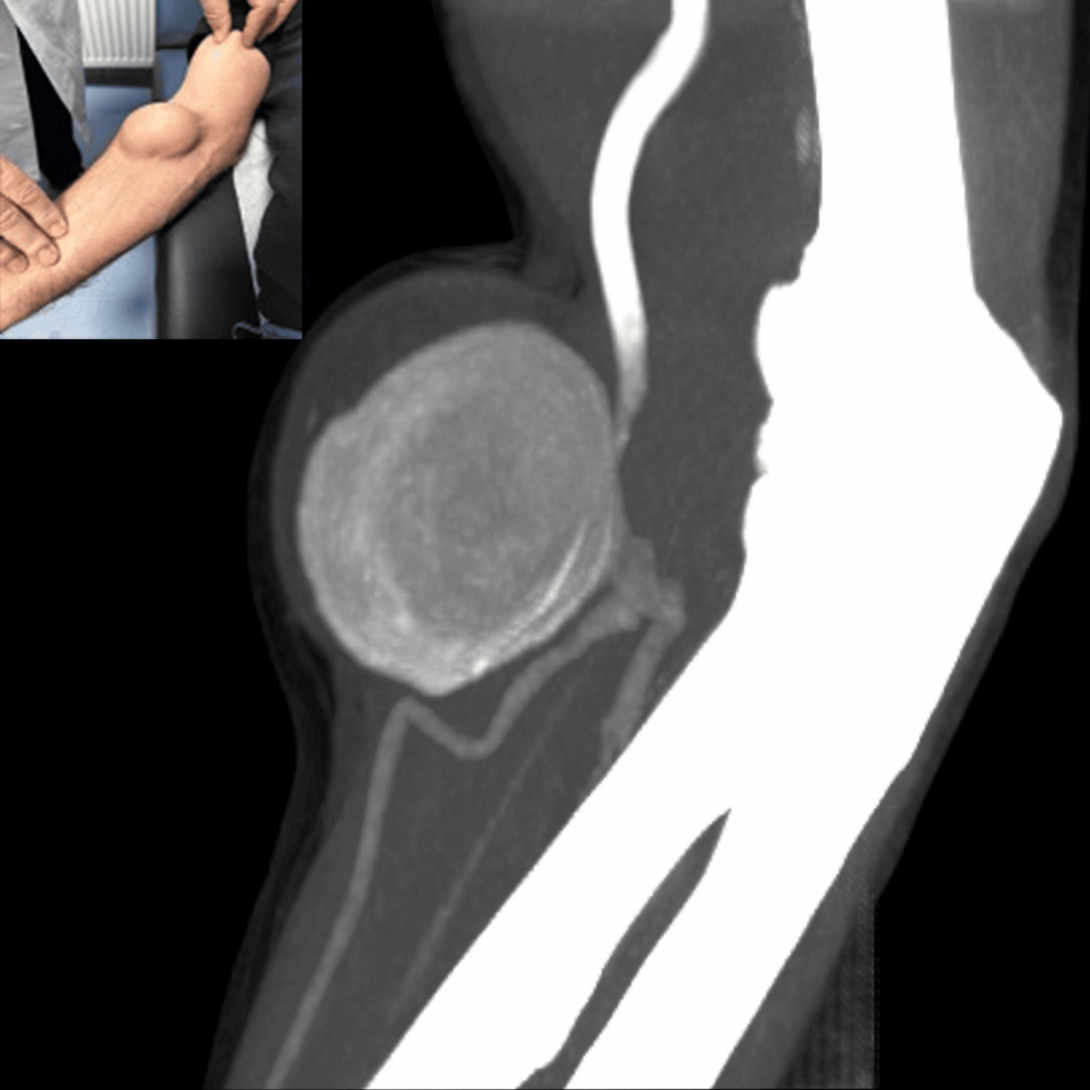
Brachial artery aneurysm of the right proximal forearm: clinical presentation, and preoperative computed tomography angiography – maximum intensity projection

A 68-year-old man, a farmer, presented to the outpatient department of our hospital with a large, slightly painful, focal swelling in the proximal part of the dominant, right forearm. Clinical evaluation revealed a giant, palpable, tender, non-compressible, pulsatile mass, located at the antecubital fossa of the proximal, volar aspect of the dominant, right forearm that was not reversible with limb elevation (Figure 1). He first witnessed the mass approximately five years ago, and, since then, it has gradually enlarged. Interestingly, he reported a blunt trauma by a heavy object in that region during agricultural activities, which had happened about six months earlier. Initially, he was evaluated by an orthopedic surgeon, who reassured him that it was merely a ganglion cyst. He was completely asymptomatic until six months prior to his visit, when he started to experience mild, local pain over the mass. Upon physical examination, there was no distal limb motor weakness or paresthesia, suggesting medial nerve compression. Moreover, there were no signs of distal limb and hand ischemia, like skin color or temperature changes in the fingertips, suggesting thromboembolic events. He was a tobacco smoker (50 pack-years) and had a past medical history of arterial hypertension, under treatment with a combination of angiotensin-converting enzyme inhibitor and calcium channel blocker. There was no history of fever, drug abuse, or invasive procedures via the upper extremity, such as arterial lines, dialysis access, or cardiac catheterization. Family history was negative for aneurysmatic, genetic, or systemic inflammatory diseases.

**Figure 2:**
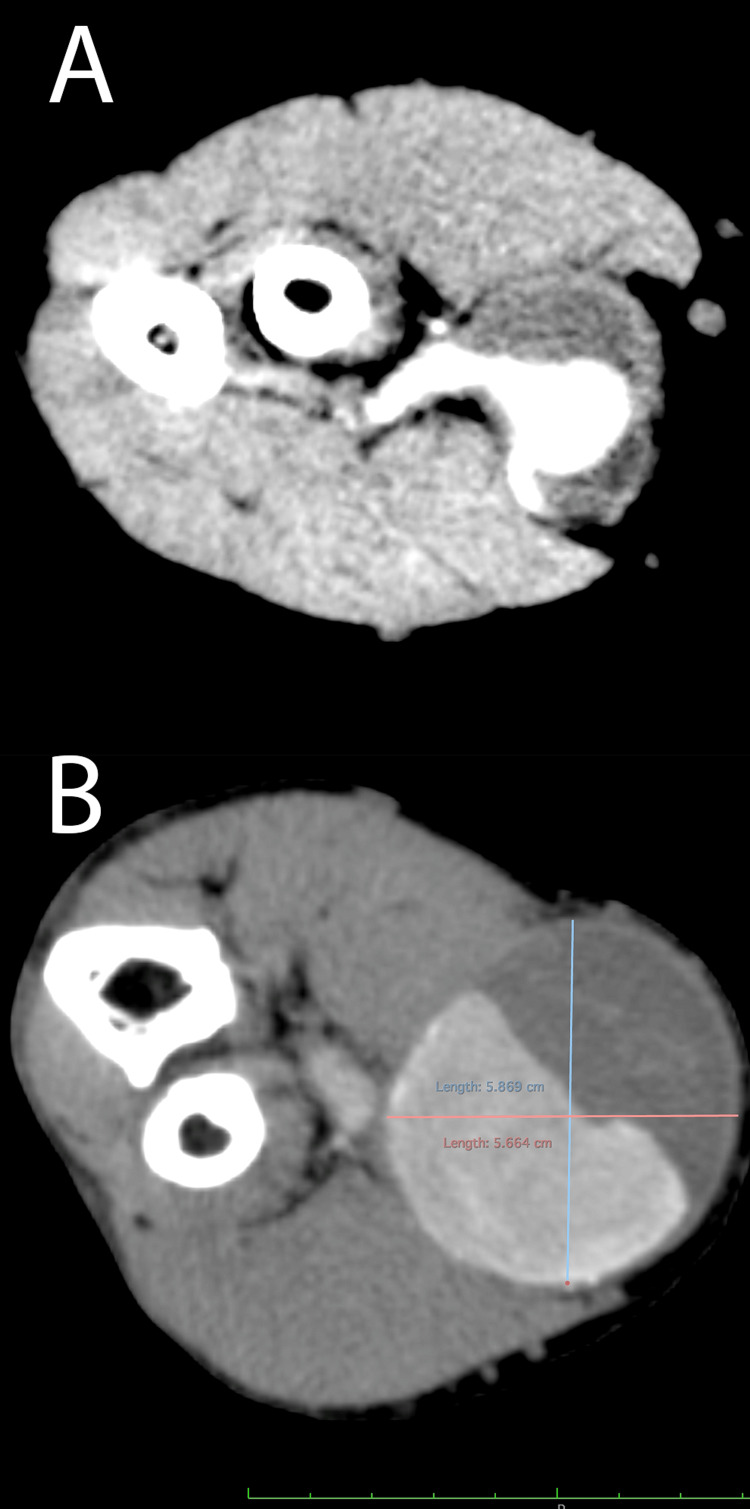
Preoperative computed tomography angiography, depicting (A) the saccular shape of the brachial artery aneurysm and (B) the maximum sac dimensions of the brachial artery aneurysm

First, we performed a thorough whole-body arterial examination and four-limb blood pressure measurements, which, apart from the aforementioned clinical sign, were normal, without clinical evidence of aortic or peripheral occlusive or aneurysmatic disease. The Allen test and ankle-brachial index were bilaterally normal. We also performed a focused colored duplex ultrasound (cDUS) of the right upper extremity, which revealed a saccular aneurysm, with turbulent flow, originating from the trunk of the right brachial artery. Then, the patient underwent a computed tomography angiography (CTA) of his trunk, neck, and both upper and lower extremities, which revealed a right saccular BAA and ruled out aortic or other peripheral aneurysmatic disease (Figure 2A). The BAA was located at the level of the right elbow, just proximal to the bifurcation of the right brachial artery, with normal imaging of both radial and ulnar arteries. The maximum dimensions of the aneurysm sac were 58x55mm, with significant intraluminal thrombus (Figure 2B).

Due to the giant size of the aneurysm sac and the presence of symptoms, the patient was offered the choice of open surgical excision, to which he gave his written informed consent, and, thus, was scheduled for surgery. Upon admission, he underwent a routine preoperative clinical, laboratory, and cardiac work-up. We also performed a bilateral ultrasound evaluation of the great saphenous vein (GSV) to determine if it was suitable for conduit. Under general anesthesia, an “S” shaped incision was performed at the level of the right antecubital fossa, extending both to the distal arm and proximal forearm. The aneurysm sac, the proximal brachial artery, and both radial and ulnar arteries were dissected free from the surrounding tissues. The patient underwent surgical excision of the aneurysm sac and arterial reconstruction of the brachial artery with end-to-end, spatulated interposition of a reversed GSV graft, which was harvested from the right lower leg [4]. After completion of the anastomoses, good arterial flow was evident through the graft, with palpable radial and ulnar pulses and triphasic Doppler signals in the palmar arch and digital arteries. The patient’s postoperative course was uneventful, and he was discharged on the third postoperative day, under single antiplatelet therapy (acetylsalicylic acid 100mg daily) and statin (atorvastatin 40mg daily), with medical recommendation to quit smoking. The pathology report confirmed the true nature of the BAA, with aneurysmal degeneration and involvement of all three vessel wall layers.

**Figure 3:**
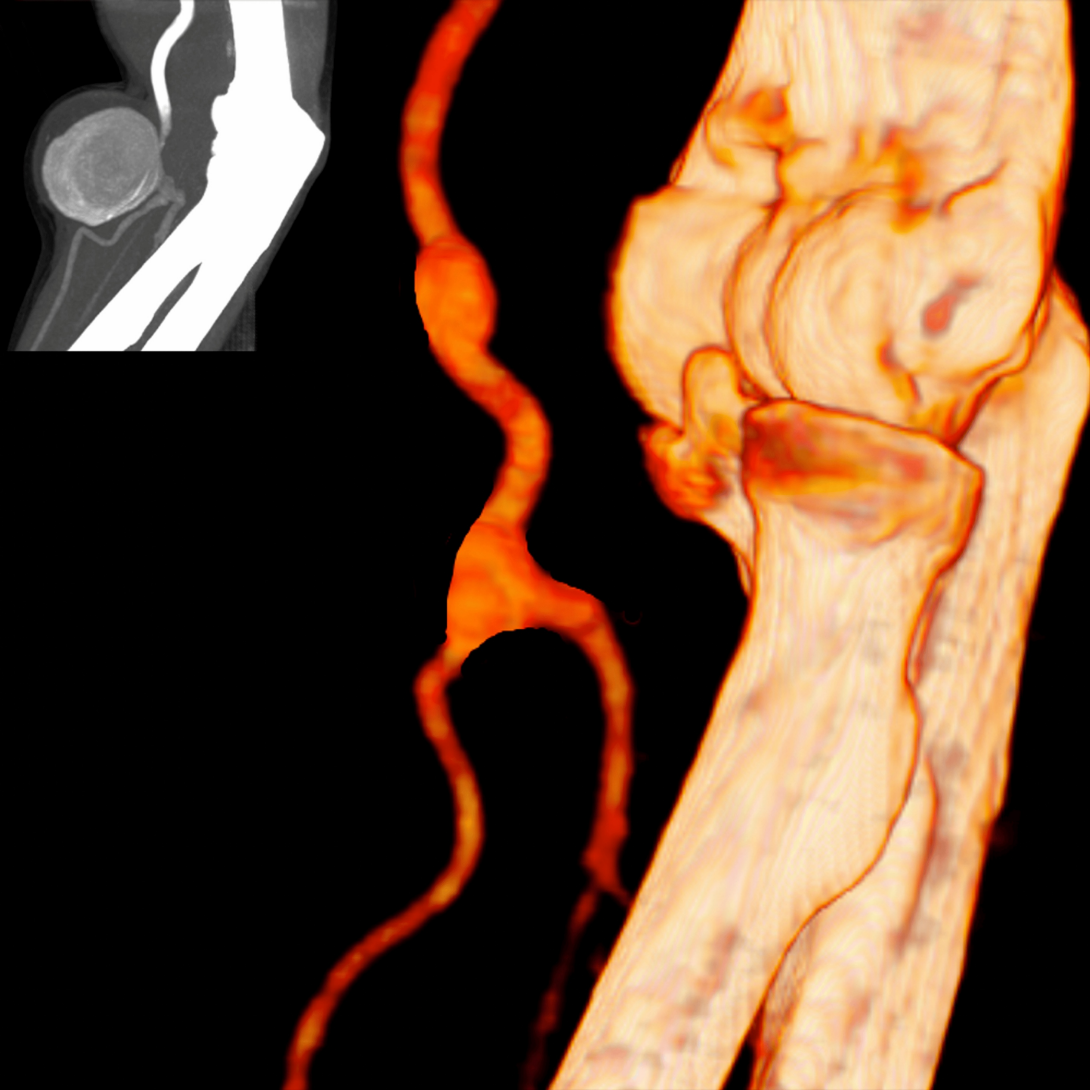
12-month follow-up computed tomography angiography 3D reconstruction, showing the arterial reconstruction after surgical excision of the brachial artery aneurysm

The patient is under a strict postoperative follow-up protocol that involves clinical and ultrasound evaluation on the first postoperative month, and every six months thereafter. Good inflow, graft flow, and outflow were noted in each visit, without ultrasound evidence of neointimal hyperplasia. Moreover, we performed a postoperative CTA one year after the surgery, confirming the good patency of the arterial reconstruction, without stenosis or anastomotic aneurysm formation (Figure 3). The patient remains up to date, two years later, free of symptoms, with normal perfusion of the right hand.

## Discussion

Upper extremity aneurysms account for less than 1% of all peripheral artery aneurysms [5]. Nearly half of them involve the brachial artery [5]. The vast majority of BAAs are pseudoaneurysms, due to local inflammation, penetrating or blunt trauma to the relatively superficially located brachial artery [6]. Moreover, in the last decades, we have witnessed a significant rise in the total number of invasive procedures via the brachial access, such as arterial lines, dialysis access, cardiac catheterization, or complex endovascular aortic aneurysm repair, which account for the high prevalence of iatrogenic false BAAs [7]. True BAAs, on the other hand, are extremely rare, comprising only 0.17% of all peripheral artery aneurysms [3]. A recent systematic review identified 113 articles, mostly case reports, reporting 157 BAAs, with a mean diameter of 36.2 ± 17.5mm. Of them, 73% were males, and the mean age was 43.1 ± 23.4 years [3]. In our case, the maximum aneurysm sac diameter was 58mm, one of the largest reported in literature.

Regarding etiology, true BAAs are either idiopathic or associated with endocarditis (due to septic heart emboli), atherosclerotic, genetic, or systemic inflammatory diseases, such as vasculitides [8]. Furthermore, they can arise as local arterial wall remodeling of an arteriovenous fistula for long-term hemodialysis access [8,9]. Finally, blunt trauma to the brachial artery can lead to the formation of a secondary true BAA, especially in chronic situations or in certain occupational or athletic settings, such as crutch users, construction workers, martial artists, or archers [8,10]. These BAAs are usually located in the distal humerus region, where the brachial artery is relatively superficial and can be compressed by the external force against the underlying bony structures [11]. This blunt trauma can lead to microscopic damage to the arterial wall, particularly the internal elastic lamina and media, followed by chronic inflammation and wall remodeling, mediated by matrix metalloproteinases [11]. Over time, it can lead to progressive wall thinning and dilatation and, finally, true aneurysm formation [11]. The aneurysm may form months or years after the initial trauma, making the causal relationship less obvious [11]. In our particular case, the patient was a farmer who constantly used his arms, especially his right, dominant hand, in his daily agricultural activities. Six months before he first witnessed the aneurysm mass in his right forearm, he had an acute blunt injury in that region, caused by direct impact with a heavy object. Over the following five years, the aneurysm gradually got larger and larger in size until it became symptomatic. So, it could be stated that this is a case of secondary, post-traumatic true BAAs.

Clinically, patients with BAAs are frequently symptomatic and can present with a visible or palpable, pulsatile mass, local pain or tenderness, or even distal limb motor weakness or paresthesia, due to medial nerve compression by the aneurysm sac [3,12]. Moreover, they can be complicated by thromboembolic events, such as distal microembolization, leading to ischemic digital lesions, or aneurysm sac thrombosis, leading to acute limb ischemia [3,13]. Rarely, the aneurysm sac can rupture, resulting in hypovolemic shock or compartment syndrome [3]. In our case, the patient was completely asymptomatic until six months prior to his visit, when he started to experience only mild, local pain over the mass, without distal limb neurological or ischemic symptoms. Differential diagnosis should be made between BAAs and various vascular malformations, benign or malignant soft tissue tumors, and mycotic abscess/pseudoaneurysm, especially in intravenous drug users [5]. In our case, the patient was initially evaluated by an orthopedic surgeon and was misdiagnosed as having merely a ganglion cyst. This highlights the need for meticulous clinical evaluation and proper differential diagnosis, as it can lead to the delayed diagnosis of a BAA, with potentially severe complications.

A high index of suspicion coupled with thorough history and clinical examination is frequently enough to diagnose a BAA, as in our case [5]. However, the definite diagnosis is made with the use of cDUS, CTA, magnetic resonance angiography (MRA) or even invasive selective angiography [14]. cDUS is considered the first-line imaging modality, due to its non-invasiveness, availability, and ability to provide both anatomic and hemodynamic information [15]. The typical appearance of a BAA in cDUS is a hypoechoic, focal, fusiform or saccular dilatation of the brachial artery, with mural thrombus, and often with turbulent flow within the aneurysm sac [5]. In most cases, cDUS is enough to differentiate between true and false BAAs and can reliably assess the location, size, anatomic relations or presence of intraluminal thrombus [5,15]. Furthermore, it can effectively monitor sac progression or the postoperative patency of the arterial reconstruction [15]. Further detailed, high-resolution imaging modalities, such as CTA or MRA, are essential when planning a surgical or endovascular intervention, in order to delineate the extent of the aneurysm, to assess the sites of vascular occlusion, in cases complicated by thromboembolism, and to determine whether anatomic variants are present, which might affect the reconstruction [3,15].

As in all cases with peripheral artery aneurysms, the diagnosis of a BAA should prompt investigation for other locations of aneurysmatic disease, in particular the aorta or other peripheral arteries [3]. Studies have reported that 20-50% of patients with BAAs have concurrent aneurysms elsewhere; most commonly abdominal aortic aneurysm, iliac, popliteal or subclavian artery aneurysm [8,16,17]. Therefore, a systematic vascular screening of the trunk, neck and both upper and lower extremities is deemed essential and should be carried out in all cases [5]. In our case, CTA was chosen to be used as the imaging modality for the whole body, due to its objectiveness and its fast and easy application, allowing for "one-stop-shop" assessment of the whole arterial system at the same time. However, meticulous ultrasound evaluation could have been equally used by experienced users.

The decision to treat or not to treat a true BAA depends on many factors, and, currently, no optimal treatment strategy exists for this rare vascular pathology [5]. Factors such as the size of the aneurysm, load of thrombus in the aneurysm sac, and the presence of symptoms or thromboembolic complications can efficiently guide the decision for surgical treatment [3]. Small, asymptomatic aneurysms can be managed by clinical and ultrasound surveillance alone [17]. However, it is impossible to predict which aneurysms are more likely to become symptomatic and lead to complications [18]. Therefore, because of the unknown natural history of BAAs, the high incidence of symptoms and/or complications, and the minimal morbidity associated with operative treatment, many authors believe that aneurysm repair should be offered to all patients with BAAs, and we generally agree with that approach [3,18]. In our case, the BAA was both giant and symptomatic, so the patient was offered the choice of open surgical excision, to which he gave his written informed consent. Moreover, due to its enormous size and its unpredictable nature, we decided to treat it urgently, scheduling the patient for surgery one week after the initial diagnosis.

Open surgical repair is the gold standard and preferred method of treatment by the vast majority of vascular surgeons [3,5,14,15]. It can be performed under local, regional, or general anesthesia, and usually consists of aneurysm sac resection and arterial reconstruction with either end-to-end primary repair or interposition grafting [3,14,15]. Usually, the preferred graft is the reversed GSV, as in our case, followed by other venous grafts, such as cephalic or basilic vein, if there isn’t a matter of size mismatch, as in our case [3,5]. The diameter of the GSV should be at least 3mm, and vein harvesting is usually performed proximally [3,5]. Synthetic grafts are the last choice for revascularization [3]. This open approach has been shown to have excellent clinical outcomes with resolution of symptoms, minimal morbidity, and good long-term results [3]. Postoperative complications are infrequent and account for only 10% of total cases, with the most often being anastomotic aneurysm formation [3]. Recently, due to the minimally invasive trend in treatment and the evolving endovascular technology, there have been some reports about endovascular repair of BAAs by the use of stent-grafts or coil embolization in high-risk patients, with a good success rate [19,20]. However, these techniques are still not in common practice.

Regarding postoperative medical treatment, since no particular guidelines for BAAs exist, we followed the general aspects of good practice guidelines for aneurysmatic disease in general. So, the patient was discharged under single antiplatelet therapy and statin, with medical recommendation to quit smoking. Finally, extensive postoperative follow-up is absolutely essential for determining the long-term results of this unusual vascular pathology. According to our common practice for patients with aneurysmatic disease, the patient is under a strict postoperative follow-up protocol, which involves clinical and ultrasound evaluation on the first postoperative month, and every six months thereafter. A CTA was performed at 12 months and will be repeated in the future if needed or dictated by clinical or ultrasound evidence.

## Conclusions

True BAAs are extremely rare, usually symptomatic, and have been associated with devastating complications, such as thromboembolic events. The current case is one of the few published cases of a giant, secondary, true BAA. Operative treatment, even in asymptomatic patients with true BAA, is advised due to the unpredictable nature, high complication rate, and low postoperative morbidity. Open surgery remains the gold standard because it is a relatively easy procedure, due to the superficiality of the brachial artery and its long-term durability. More research into the pathophysiology of this rare pathology is needed to explore the risk factors and further understand its natural history.

## References

[1] Johnston KW, Rutherford RB, Tilson MD, Shah DM, Hollier L, Stanley JC (1991). Suggested standards for reporting on arterial aneurysms. Subcommittee on Reporting Standards for Arterial Aneurysms, Ad Hoc Committee on Reporting Standards, Society for Vascular Surgery and North American Chapter, International Society for Cardiovascular Surgery. J Vasc Surg.

[2] Schunn CD, Sullivan TM (2002). Brachial arteriomegaly and true aneurysmal degeneration: case report and literature review. Vasc Med.

[3] Alam SM, Moin S, Gilani R, Jawad N, Abbas K, Ellahi A (2024). True brachial artery aneurysm: a systematic review. J Pak Med Assoc.

[4] Pitoulias GA, Donas KP, Bisdas T, Stavroulakis K, Christopoulos DC (2015). Treatment of symptomatic popliteal artery aneurysms with venous bypass by the AESA (asymmetric end-to-end spatulated anastomosis) technique. Cor et Vasa.

[5] Shaban Y, Elkbuli A, Geraghty F, Boneva D, McKenney M, De La Portilla J (2020). True brachial artery aneurysm: a case report and review of literature. Ann Med Surg (Lond).

[6] Kemp K, Radwan R, Shingler G, Davies C (2014). Brachial artery pseudoaneurysm. BMJ Case Rep.

[7] Tamanaha Y, Sakakura K, Taniguchi Y (2019). Comparison of postcatheterization pseudoaneurysm between brachial access and femoral access. Int Heart J.

[8] Saidman AY, Perler BA (2023). Upper extremity aneurysms. Rutherford’s Vascular Surgery and Endovascular Therapy, 10th.

[9] Eugster T, Wigger P, Bölter S, Bock A, Hodel K, Stierli P (2003). Brachial artery dilatation after arteriovenous fistulae in patients after renal transplantation: a 10-year follow-up with ultrasound scan. J Vasc Surg.

[10] Simsek B, Guclu O, Huseyın S, Yuksel V (2021). Bilateral brachial artery aneurysms with distal embolisms in a patient with prolonged crutch compression. Vasc Specialist Int.

[11] Agarwal R, Sharma P, Gupta PC, Atturu G (2019). True brachial artery aneurysm associated with 44-year-old non-united supracondylar fracture - a case report and literature review. Eur J Vasc Endovasc Surg.

[12] Gray RJ, Stone WM, Fowl RJ, Cherry KJ, Bower TC (1998). Management of true aneurysms distal to the axillary artery. J Vasc Surg.

[13] Grande-Garcia R, Anaya-Ayala JE, Barragán-Galindo L (2024). Ischemic complication of a rare traumatic true brachial artery aneurysm: a case report. Vasc Specialist Int.

[14] Igari K, Kudo T, Toyofuku T, Jibiki M, Inoue Y (2013). Surgical treatment of aneurysms in the upper limbs. Ann Vasc Dis.

[15] Gonzalez-Urquijo M, Marine L, Vargas JF, Valdes F, Mertens R, Bergoeing M, Torrealba J (2022). True idiopathic brachial artery aneurysm treated with a saphenous vein graft. Vasc Endovascular Surg.

[16] Hudorović N, Lovričević I, Franjić DB, Brkić P, Tomas D (2010). True aneurysm of brachial artery. Wien Klin Wochenschr.

[17] Senarslan DA, Yildirim F, Tetik O (2019). Three cases of large-diameter true brachial and axillary artery aneurysm and a review of the literature. Ann Vasc Surg.

[18] Dawson J, Fitridge R (2013). Update on aneurysm disease: current insights and controversies: peripheral aneurysms: when to intervene - is rupture really a danger?. Prog Cardiovasc Dis.

[19] Yiğit G, Özen S, Özen A, İşcan HZ (2019). Isolated brachial artery aneurysm successfully treated with a covered stent in a patient with Behçet's disease. Turk Gogus Kalp Damar Cerrahisi Derg.

[20] Yılmaz F, Güvendi Şengör B, İzci S (2021). Successful management of a brachial artery aneurysm with percutaneous intervention and one-month rivaroxaban therapy. Anatol J Cardiol.

